# Susceptible and mCry3A resistant corn rootworm larvae killed by a non-hemolytic *Bacillus thuringiensis* Cyt1Aa mutant

**DOI:** 10.1038/s41598-018-36205-6

**Published:** 2018-12-13

**Authors:** Alejandra Bravo, Jazmin A. López-Diaz, Takashi Yamamoto, Kathleen Harding, Jian-Zhou Zhao, Gretel Mendoza, Janette Onofre, Mary-Carmen Torres-Quintero, Mark E. Nelson, Gusui Wu, Amit Sethi, Mario Soberón

**Affiliations:** 1 Instituto de Biotecnología, Universidad Nacional Autónoma de México. Apdo. Postal 510-3, Cuernavaca, 62250 Morelos Mexico; 2 DuPont Pioneer, Hayward, CA 94545 USA; 3 DuPont Pioneer, Johnston, IA 50131 USA

## Abstract

The western corn rootworm (WCR) *Diabrotica virgifera virgifera* causes substantial damage in corn. Genetically modified (GM) plants expressing some *Bacillus thuringiensis* (Bt) insecticidal Cry proteins efficiently controlled this pest. However, changes in WCR susceptibility to these Bt traits have evolved and identification of insecticidal proteins with different modes of action against WCR is necessary. We show here for the first time that Cyt1Aa from Bt exhibits toxicity against WCR besides to the dipteran *Aedes aegypti* larvae. Cyt1Aa is a pore-forming toxin that shows no cross-resistance with mosquitocidal Cry toxins. We characterized different mutations in helix α-A from Cyt1Aa. Two mutants (A61C and A59C) exhibited reduced or absent hemolytic activity but retained toxicity to *A*. *aegypti* larvae, suggesting that insecticidal and hemolytic activities of Cyt1Aa are independent activities. These mutants were still able to form oligomers in synthetic lipid vesicles and to synergize Cry11Aa toxicity. Remarkably, mutant A61C showed a five-fold increase insecticidal activity against mosquito and almost 11-fold higher activity against WCR. Cyt1Aa A61C mutant was as potent in killing WCR that were selected for resistance to mCry3A as it was against unselected WCR indicating that this toxin could be a useful resistance management option in the control of WCR.

## Introduction

The western corn rootworm (WCR) *Diabrotica virgifera virgifera* is an insect pest that attacks cornfields eating the roots of the plants causing extensive damage. It is principally found in North America and Europe resulting in grower-losses in excess of one billion dollars per year^1^. In addition, this pest is highly adaptable and has evolved resistance to chemical insecticides^2^. Genetic modified (GM) plants expressing toxins from *Bacillus thuringiensis* (Bt) such as Cry3Bb1, mCry3A, eCry3.1Ab and Cry34/35Ab1 have been used in the control of this pest in cornfields^3–5^. However, changes in WCR susceptibility to these traits have evolved in some areas potentially compromising the effectiveness of GM plants^6,7^. In addition, resistance to Cry3Bb showed cross-resistance to mCry3A and eCry3.1Ab toxins further highlighting the challenges facing Bt-corn technology in the control of this pest^8,9^.

WCR resistance to Bt-corn has encouraged researchers to look for additional strategies and novel proteins that could be used to control this challenging pest. Recently new proteins that kill WCR have been identified from non-Bt bacteria such as the small IPD072Aa protein from *Pseudomonas chlororaphis* or the PIP-47Aa protein from *P*. *mosselii*^10,11^. These novel toxins do not share receptors with Cry3A or Cry34/35Ab1 and neither showed cross-resistance in Cry3A-selected insects^10,11^, while IPD072Aa also showed no cross-resistance to Cry34/35Ab1-selected insects^10^. In addition a binary toxin named AflP-1A/1B isolated from the bacteria *Alcaligenes faecalis* was shown to be toxic against WCR although it shares a similar mode of action as the Cry34/35Ab1 toxin since WCR population resistant to Cry34/35Ab1 showed cross-resistance to AflP-1A/1B^12^.

Here we show that Cyt1Aa, previously characterized as a mosquitocidal toxin, display moderate toxicity against WCR. Under sporulation conditions Bt produces two main types of insecticidal toxins named Cry or Cyt, which are toxic to different insect orders^13^. The Cyt toxins are composed of a single α−β domain with seven to eight β-strands wrapped by α-helices^14–17^. Cyt1Aa toxin is mostly active against dipteran larvae^18^ and it is found in Bt subspecies *israelensis* strain along with the mosquitocidal Cry4Aa, Cry4Ba and Cry11Aa toxins. Besides its insecticidal activity, Cyt1Aa toxin has cytolytic activity against red blood cells^19^ and this hemolytic activity could limit its applications in GM products. The Cyt proteins form high molecular weight oligomers, which insert into the membrane of the midgut targeted-cells forming lytic pores^20–23^. It has been proposed that β5-β7 region of Cyt1Aa is likely involved in Cyt1Aa membrane insertion while helices α-A and α-C are involved in Cyt1Aa oligomerization and recognition of membrane lipids^14,23–26^. It was shown that oligomerization of Cyt1Aa and Cyt2Aa toxins is a key step in membrane binding and pore formation since certain mutations in helix α-C affected oligomerization and membrane insertion, reducing their toxicities against *Aedes aegypti* larvae as well as their hemolytic activities^23,27^.

One of the most interesting features of Cyt1Aa is its capacity to synergize the toxicity of different Cry toxins such as Cry11Aa or Cry4Ba^28–30^. Moreover Cyt1Aa is able to overcome resistance of *Culex quinquefasciatus* to Cry4Aa, Cry4Ba and Cry11Aa toxins^31^. It has been proposed that Cyt1Aa functions as a receptor of Cry11Aa since binding of this toxin to Cyt1Aa facilitates oligomer formation of Cry11Aa and its membrane insertion^29,32^.

To determine the role of Cyt1Aa helix α-A in the mode of action of this toxin, different residues were mutated and these mutants were analyzed for oligomerization, synergism with Cry11Aa, as well for the hemolytic and insecticidal activities against dipteran and coleopteran larvae. Our results show that two mutations located in helix α-A of Cyt1A reduced hemolysis of red blood cells, but did not alter oligomerization, synergism with Cry11Aa, or toxicity against *A*. *aegypti* or WCR larvae. Moreover, one of these mutants showed a significant increase in its insecticidal potency against both mosquitoes and WCR larvae and also displays toxicity against the WCR population resistant to mCry3A. These data indicate that this non-hemolytic Cyt1Aa toxin with increased toxicity to WCR might have the potential for efficient WCR control in transgenic corn.

## Results

### Insecticidal activity of Cyt1Aa against the coleopteran pest

**Figure 1:**
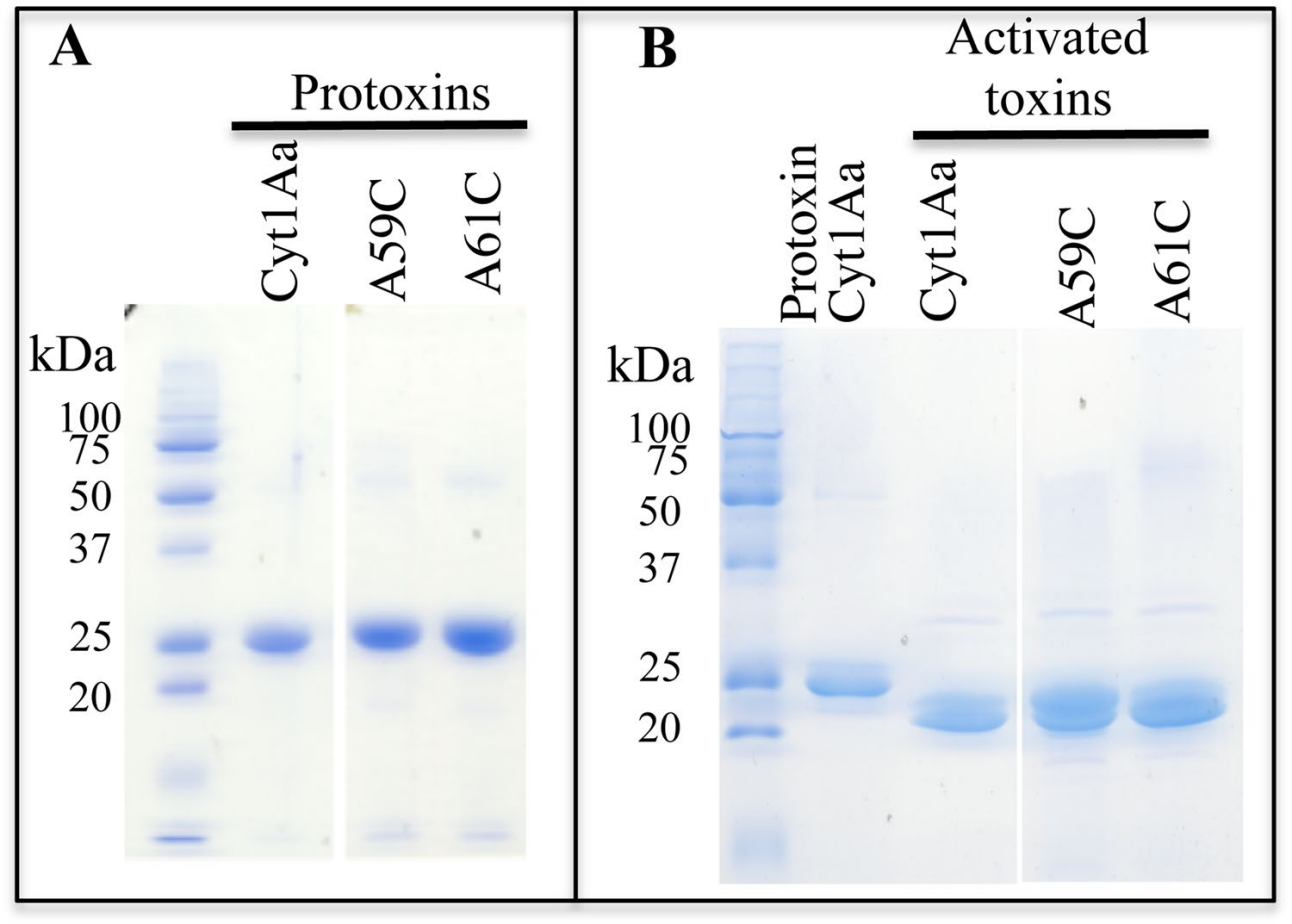
SDS-PAGE analysis of the Cyt1A wild type and mutant proteins produced in *B*. *thuringiensis*. Panel A, Protoxins solubilized from purified crystal inclusions. Panel B, trypsin activated toxins. All samples were boiled 5 min before loading into the SDS-PAGE and the gel was stained with coomassie brilliant blue.

Previous report showed the potential toxicity of Cyt1Aa against coleopteran species^34^. The toxicity of Cyt1Aa was evaluated against WCR larvae in bioassays as described in Methods. The evaluation of the mean growth inhibition concentration (IC_50_) value was performed with solubilized protoxin proteins (Fig. 1A) that were obtained from crystal inclusions purified by sucrose gradients as reported in Methods. The control included the same volume of solubilization buffer. These bioassays showed that Cyt1Aa has an IC_50_ of 335 μg per cm^2^ of diet against neonate WCR larvae.

### Isolation of Cyt1Aa α-A mutants

To determine the potential role of Cyt1Aa helix α-A (^49^PNYILQAIMLANAFQNAL^66^) in Cyt1Aa mode of action, the amino acid residues L58, A59, A61 and F62, located in the hydrophobic phase of the helix, were mutated as described in Materials and Methods. The plasmids containing the Cyt1Aa mutant genes were transformed into acrystalliferous Bt407 strain and protein expression was analyzed under sporulation condition. Mutants A59E and F62R did not produce the corresponding protoxin, while L58E mutant was produced at much lower levels compared to Cyt1Aa. However, after solubilization of protein crystals by alkaline treatment, L58E protein was not soluble (data not shown). Therefore A59E, F62R and L58E mutants were not further analyzed. In contrast A59C and A61C mutants were produced as 27 kDa proteins upon sporulation (Fig. 1A). These proteins were solubilized and treated with trypsin for toxin activation. Figure 1B shows that, as Cyt1Aa, A59C and A61C yielded a 22 kDa activated protein after trypsin treatment indicating no major structural changes caused by these mutations.

### Insecticidal and hemolytic activities of Cyt1Aa α-A mutants

Table 1 shows the mean lethal concentration (LC_50_) values of the toxicity data against *A*. *aegypti* and the mean growth inhibition concentration (IC_50_) values in WCR. The Cyt1Aa A59C mutant display two-fold lower toxicity against *A*. *aegypti* compared to Cyt1Aa, while A61C showed five-fold higher toxicity to this mosquito larvae (Table 1). Bioassay data against WCR showed that A59C displayed five-fold higher toxicity to WCR while A61C mutant showed 11-fold higher toxicity against WCR. The wild type Cyt1Aa and A59C mutant were not active enough against the mCry3Aa resistant WCR^33^ population to evaluate cross-resistance accurately (data not shown). In contrast, Cyt1Aa A61C mutant showed sufficient activity against the mCry3A-resistant WCR (IC_50_ = 131 μg/cm^2^ diet, with 95% CL of 68.5–1149 μg/cm^2^ diet) to reveal no significant cross-resistance (RR = 2) when compared to the activity against susceptible WCR (IC_50_ = 65.5 μg/cm^2^ diet, with 95% CL of 41.59–127.5 μg/cm^2^ diet) when tested at the same time.

**Table 1:** Insecticidal activity of Cyt1Aa mutants against 4^th^ instar *Aedes aegypti* larvae and neonate WCR larvae.

Toxin	LC_50_^a^ (ng/ml) against *Aedes aegypti*	IC_50_^b^ (μg/cm^2^) against control WCR
Cyt1Aa	1100 (880–1480)^c^	335.6 (284.8–399.2)
A59C	2419 (1861–3653)	63.2 (38.8–96.5)
A61C	212 (131–273)	29.2 (24.8–35.1)
Cry11Aa	669 (476–994)	ND^d^

^a^Mean lethal concentration value.

^b^Mean growth inhibition concentration value.

^c^95% confidential limits calculated by Probit statistical analysis within the parenthesis.

^d^Not determined.

**Figure 2:**
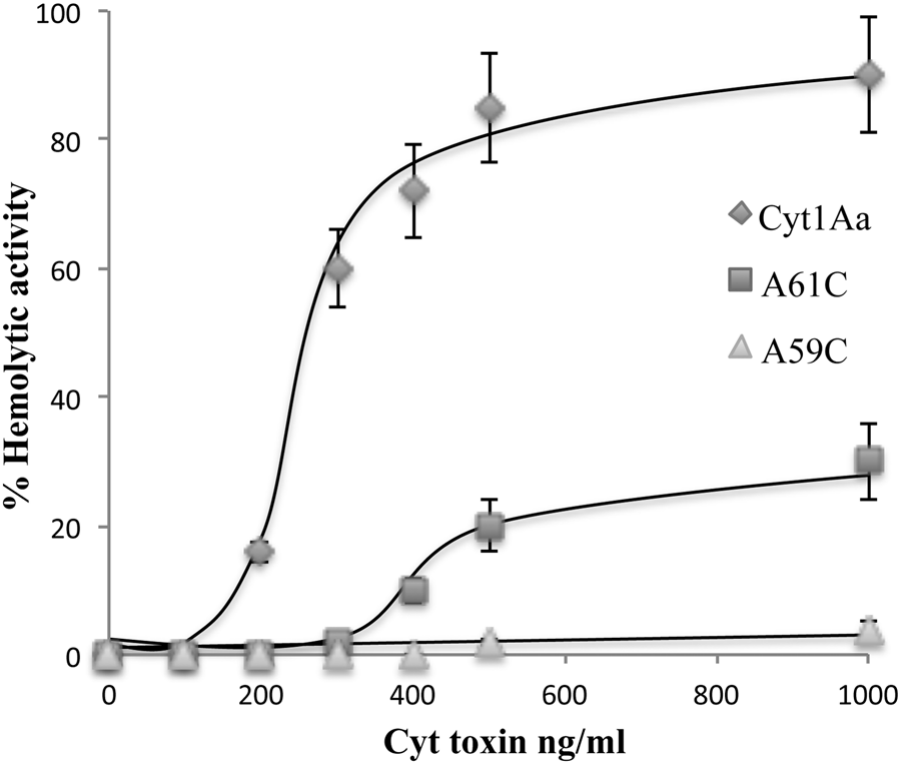
Hemolytic activity of Cyt1A wild type and mutant proteins assayed in Rabbit red blood cells. Positive control showing 100 percent hemolysis was defined after incubation of the same volume of rabbit red blood cells with dechlorinated H_2_O. Negative controls were red blood cells incubated with buffer A. These assays were performed three times in triplicate each time. Standard deviations are shown in the figure.

We determined the hemolytic activity of Cyt1Aa and helix α-A mutants. Rabbit red blood cells were incubated with increasing concentrations of trypsin-activated toxins. Figure 2 shows that both α-A mutants were severely affected in hemolysis since wild type Cyt1Aa toxin showed a fifty percent effective dose (ED_50_) of 250 ng/ml while mutant A61C lysed only 30% of the red blood cells with 1000 ng/ml. A59C exhibited no hemolytic activity at the highest toxin concentration tested (Fig. 2).

The capacity of Cyt1Aa and α-A mutants to synergize Cry11Aa toxicity to *A*. *aegypti* larvae was determined. As described in Methods we prepared mixtures of Cyt1Aa and Cry11Aa that would give a toxicity of 20% based on their corresponding LC_50_ toxicity values. Table 2 shows that A59C and A61C synergize the activity of Cry11Aa since the toxicity of the protein mixtures showed a three- to four-fold higher toxicity than the expected mortality.

**Table 2:** Analysis of synergism of Cyt1Aa or mutant toxins with Cry11Aa toxin in 4^th^ instar *Aedes aegypti* larvae.

Toxin	S_(*toxin*)OBS_^a^ = (Rep1 + Rep2 + Rep3)/n	S_(*Cyt1Aa*, *Cry11Aa*)EXP_^b^ = S_(*Cyt1Aa*)OBS_ × S_(*Cry11Aa*)OBS_	Expected mortality^c^ = (1 − S_(*Cyt1Aa*, *Cry11Aa*)EXP_) × 100%	Observed mortality^d^_*Cyt1Aa*+*Cry11Aa*_
Cyt1Aa	1.00	0.80	20%	90 ± 10%
A59C	1.00	0.80	20%	57 ± 20%
A61C	0.93	0.75	25.3%	83 ± 15%
Cry11Aa	0.80			

^a^Observed survival of individual toxin S_(*toxin*)OBS_ corresponds to the observed proportion of larvae that survived to the exposure to Cyt1Aa or mutant toxins. Observed mortality was 20% with Cry11Aa at 200 ng per ml and 0% with Cyt1Aa at 75 ng Cyt1Aa per ml. n = 30 larvae for each toxin tested.

### Effect of Cyt1Aa A59C and A61C on toxin oligomerization

**Figure 3:**
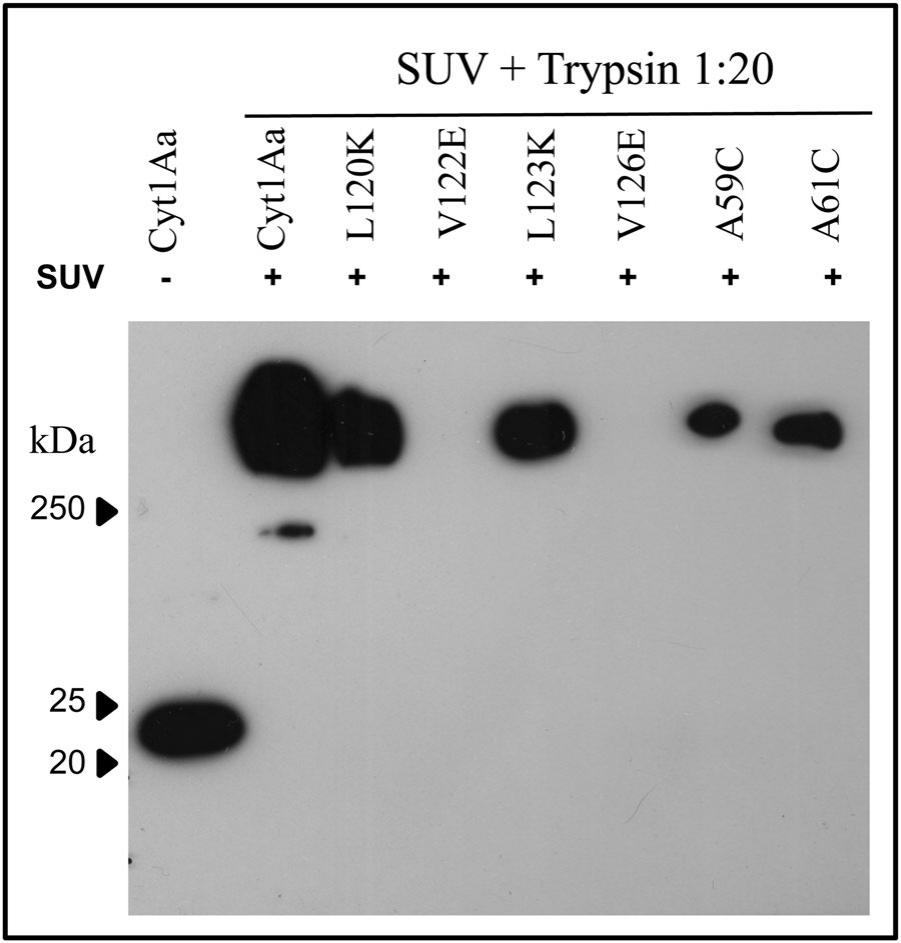
Oligomerization of Cyt1Aa and mutant proteins after activation of solubilized protoxin, with trypsin in the presence of SUV liposomes. Samples were heated 3 min at 65 °C before loading into the SDS-PAGE transferred to PVDF and reveled in western blot assay as described in materials and methods using polyclonal anti-Cyt1A antibody and goat anti-rabbit antibody coupled to horseradish peroxidase.

To determine the effect of A59C and A61C mutations on Cyt1Aa oligomerization, soluble protoxins of Cyt1Aa and of the two α-A mutants were incubated with small unilamellar vesicles (SUV) and trypsin. The vesicles were collected by centrifugation and analyzed by western blot using anti-Cyt1Aa antibody as described in Materials and Methods. As negative control we included two Cyt1Aa mutants located in helix α-C that were previously characterized to be affected in oligomerization (V122E and V126E) and two helix α-C mutants that were not affected in oligomerization (L120K and L123K)^23^. Figure 3 shows that Cyt1Aa and A59C and A61C mutants produced high molecular weight oligomers after protease activation in the presence of synthetic membranes. This was similar to the previously characterized α-C mutants L120K or L123K that were not affected in oligomer formation^23^. In contrast, helix α-C mutants V122E and V126E did not form oligomers as previously reported^23^ (Fig. 3A). These results show that Cyt1Aa A59C and A61C mutations did not affect toxin oligomerization.

## Discussion

Cyt1Aa toxin shows a specific insecticidal activity mainly to dipteran insects. However, previous reports showed that Cyt1Aa could have coleopteran activity since it was able to kill the cottonwood leaf beetle, *Chrysomela scripta*^34^. It was also shown that Cyt1Aa exhibits low toxicity against homopteran pests such as the pea aphid *Acyrthosiphon pisum*^35^. Here we show that Cyt1Aa has insecticidal activity against the agriculturally important coleopteran pest WCR, *Diabrotica virgifera virgifera* (Le Conte). In addition, Cyt1Aa shows cytolytic activity against red blood cells^19^.

It was previously suggested that Cyt1Aa helices α-A and α-C were involved in the initial binding of Cyt1Aa to membrane lipids and in toxin oligomerization^36^. In agreement with this, it was later shown that certain Cyt1Aa helix α-C mutations affected toxin oligomerization that correlated with a severe effect in its insecticidal and hemolytic activities but did not affect its synergism with Cry11Aa toxin^23^.

Here we show that Cyt1Aa mutations in helix α-A reveal amino acids (A59C and A61C) that can differentiate the lytic effects of Cyt1Aa on rabbit red blood cells from the insecticidal activity against WCR since cysteine replacement of these amino acids resulted in significant reduction of Cyt1A hemolytic activity and a significant increase in the toxicity to WCR compared to the wild type Cyt1Aa.

The Cyt1Aa mutations A59C or A61C did not affect oligomerization of the toxin with exposure to synthetic SUV. In the case of the closely related Cyt2Aa toxin, a similar mutation at A61C located also in helix α-A was affected in oligomer formation with exposure to SUV although it showed a similar toxicity to mosquito larvae^27^. We do not know the reason for this difference but different experimental conditions in the oligomerization assays might explain it. In the case of Cyt2Aa, liposomes were prepared with a lipid mixture of phosphatydylcholine: cholesterol: stearylamine in a molar ratio of 4:3:1^27^, while we prepared liposomes with the same lipid mixture but in a molar ratio of 10:3:1. Also, Cyt2Aa activated toxin was incubated with the liposomes during the oligomerization assay^27^, while we co-incubated Cyt1Aa protoxin along with trypsin (for toxin activation) in the presence of the liposomes to induce Cyt1Aa-oligomerization.

Cyt1Aa has a distinct mode of action than Cry toxins since it does not rely on protein receptors for killing mosquitoes. It has been shown that *Culex quinquefasciatus* insects resistant to Cry11Aa showed no cross-resistance to Cyt1Aa^37^ and that Cyt1Aa can overcome the resistance of the mosquito larvae to Cry4Aa, Cry4Ba and Cry11Aa toxins^31^. These data suggest that Cyt1Aa toxin could represent an interesting alternative for the control of other insect pests.

Our results show that helix α-A is involved in Cyt1Aa toxin specificity. The α-A helix A61C and A59C mutations both resulted in more toxic proteins against WCR while only the A61C mutation was significantly more toxic to *A*. *aegypti* than Cyt1Aa. The A61C mutant also showed insecticidal activity against the mCry3A resistant population of WCR. Importantly, both Cyt1Aa α-A mutants had reduced hemolytic activity with A59C exhibiting no hemolytic activity, improving its potential for use in transgenic corn for protection from damage caused by WCR. These data indicate that the α-A helix of Cyt1A plays an important role in hemolytic activity and that its insecticidal activity is independent of its hemolytic activity. These novel Cyt1Aa mutant toxins offer potential as additional alternatives to currently utilized Bt toxins for the effective control of WCR.

## Materials and Methods

### Production of Cyt1Aa and Cry11Aa Proteins

Production of Cyt1Aa and Cry11Aa proteins was done as described previously^23^. The Cyt1Aa or Cry11Aa protoxins were produced in *B*. *thuringiensis* 407 acrystalliferous strain transformed with plasmid pWF45^38^ or pCG6^39^. Cyt1Aa mutants were expressed also in the *B*. *thuringiensis* 407 acrystalliferous strain. These Bt strains were grown in solid nutrient broth sporulation medium^40^ (4 days, 30 °C), these media were supplemented with 10 μg/ml erythromycin for Cyt1Aa or 25 μg/ml erythromycin for Cry11Aa. Spores and crystals were washed with 0.3 M NaCl, 0.01 M EDTA, pH 8.0, by centrifugation (10 min, 10,000 rpm, 4 °C), washing steps were done three times. Pellets were stored at −20 °C until used. The crystal/spore pellet was suspended in 0.05% Triton X-100, 300 mM NaCl, 20 mM Tris-HCl pH 7.2, sonicated two times 1 min and the inclusion bodies were purified by sucrose gradient centrifugation as reported^41^. Cyt1A proteins were solubilized 1 h at 37 °C in 50 mM Na_2_CO_3_, 10 mM DTT, pH 10.5, agitation at 350 rpm and centrifuged for 10 min at 10,000 rpm 4 °C. The soluble protoxins were recovered in the supernatant. Protein concentrations were determined by the Bradford assay. Finally, Cyt1Aa protoxin was activated with trypsin 1:20 (Trypsin: Cyt1Aa) ratio (Sigma-Aldrich Co., St Louis, MO) w/w for 2 h at 30 °C. Supplementary Figs S1 and S2 show additional images of SDS-PAGE analysis of Cyt1Aa, A59C and A61C proteins purified from *B*. *thuringiensis*.

### Site-Directed Mutagenesis of Cyt1Aa Toxin

Mutagenesis of Cyt1Aa toxin was performed in pWF45 plasmid by using QuikChange XL Site-Directed kit (Stratagene La Jolla, CA) as previously described^23^. Table 3 shows the information of mutagenic oligonucleotides that were synthesized by Sigma-Aldrich (St Louis, MO). Mutated plasmids were transformed in *E*. *coli* X-L1 blue strain and transformants were selected in LB Ampicillin 100 μg/ml at 25 °C. Plasmid DNA was purified using a DNA extraction kit (Qiagen, Hilden, GER) and sequenced at the Institute of Biotechnology UNAM. Finally plasmids were transformed into Bt 407 strain and transformants were selected in LB erythromycin 10 μg/ml at 30 °C. We confirmed the sequence of these mutants by PCR amplification using IRE1d-IRE4r oligonucleotides (Table 3).

**Table 3:** Sequence of mutagenic oligonucleotides. The sequence of mutagenic codon is underlined and labeled with bold letters.

Oligonucleotide	DNA sequence
A59C	TTG CAA GCA ATT ATG TTA **TGT** AAT GCC TTT CAA AAT GC
A61C	GCA AGC AAT TAT GTT AGC AAA C**TG T**TT TCA AAA TGC ATT AGT TCC C
L58E	TAT ATA TTG CAA GCA ATT ATG **GAA** GCA AAT GCG TTT CAA AAT GC
A59E	TAT ATT GCA AGC AAT TAT GTT A**GA A**AA TGC GTT TCA AAA TGC
F62R	AGC AAT TAT GTT AGC AAA TGC A**CG G**CA AAA TGC GTT AGT TCC
IRE1d	TGT GAA TTC ATG GAA AAT TTA AAT CAT TG
IRE4r	CTA CTC GAG GAG GGT TCC ATT AAT AGC

### Insect bioassays

The *Aedes aegypti* mosquitoes used in this work were reared at Instituto de Biotecnologia facilities, at 28 °C, 75% humidity and a 12 h: 12 h light: dark photoperiod. Mosquitocidal bioassays were performed as described before^23^. We used 10 early 4^th^-instar larvae in 100 ml of dechlorinated water per doses. We used ten different concentrations from 50 to 10000 ng/ml of spore/crystal suspensions of each Cyt1Aa protein, wild type or mutants. These samples were previously sonicated (1 min) using an ultrasonic processor (Cole-Palmer) and diluted into the 100 ml water containers that will be used in the bioassay. The negative control was dechlorinated water. Larvae viability was examined after 24 h. Probit analyses were used to determine the mean lethal concentration (LC_50_) using statistical parameters of the data in triplicate performed in three independent assays (Polo-Plus LeOra Software).

Susceptible WCR and mCry3A resistant WCR (laboratory selected^33^) populations were reared at DuPont Pioneer, Johnston, IA headquarters. Bioassays were performed in 96-well plates. Isolated Cyt1Aa crystals were solubilized for 1 h at room temperature in 100 mM sodium bicarbonate buffer supplemented with 20 mM DTT, pH 10. Samples were centrifuged at 13,000 rpm 30 min and the supernatant was collected. The supernatant was concentrated up to 1 ml using Ultra 15 Amicon filters. Serial 2X dilutions of the solubilized protoxins were prepared with sodium bicarbonate buffer and 20 μl of each protoxin dilution was overlaid on 50 μl diet in 96-well plates. Three to five neonate susceptible WCR larvae were placed in each well and the assay plates were incubated at 25 °C for four days. For assessing cross-resistance three to five 24 h-born mCry3Aa resistant WCR larvae, that were fed 24 h on diet, were placed in each well and the assay plates were incubated at 25 °C for six days. Sodium bicarbonate buffer was used as a negative control. The growth inhibition concentration (IC_50_) defined as the concentration of protein that resulted in 50% reduction in larval development when compared to larvae feeding on diet alone was determined using Microsoft Excel from triplicate assays.

### Synergism assay

Synergism was evaluated as previously described^42^ by testing for deviation from the null hypothesis of simple independent action of the toxins. This hypothesis assumes that the surviving larvae to the exposure of mixture of toxins are the product of the surviving larvae to each toxin separately. The formula S_(*ab*)EXP_ = S_(*a*)OBS_ X S_(*b*)OBS_^42^ was used where S_(*ab*)EXP_ is the proportion of larvae expected to survive to the mixture of toxins *a* and *b*, S_(*a*)OBS_ and S_(*b*)OBS_ are the observed proportion of larvae that survived to toxin *a* or toxin *b*. We used 30 larvae per toxin or toxins mixtures. The expected mortality to the mixture of toxins *a* and *b* was obtained by using (1 − S_(*ab*)EXP_) × 100% and the expected numbers of dead and live larvae were obtained by multiplying the expected mortality and survival rates by the sample size used with each toxin. All synergism assays were done in triplicate and Fisher’s exact test was used to determine the significant differences among observe and expected mortality data.

### Hemolysis assays

Hemolytic assays were done as previously described^22^. We used red blood cells from Rabbits which were washed using buffer A (0.1 M dextrose, 0.07 M NaCl, 0.02 M sodium citrate, 0.002 M citrate, pH 7.4) three times and then diluted to 2 × 10^8^ cells/ml in the same buffer. The reaction was perfumed in a final volume of 0.2 ml in 96 wells microtiter plates, which contains 20 μl of reed blood cells and different concentrations of Cyt1Aa toxin (20–1000 ng) in the same buffer. Samples were incubated at 37 °C for 30 min, after that time were centrifuged (2,500-x *g*, 5 min, 4 °C), the supernatants were collected and absorbance at 405 nm was determined. Positive control was rabbit red blood cells with dechlorinated H_2_O showing 100% hemolysis. Negative control was red blood cells with buffer A. All assays were done three times in triplicate and *t*-test analysis were performed using GraphPad Prism statistical program.

### Preparation of Small Unilamellar Vesicles (SUV)

SUV were prepared as previously reported^23^. A mixture of lipids egg-yolk phosphatidyl choline (PC), cholesterol (Ch) (Avanti Polar Lipids, Alabaster, AL) and stearylamine (S) (Sigma-Aldrich, St Louis, MO) at 10:3:1 proportion, was used. The final concentration of the total lipid mixture was 0.65 μmol. The mixture was dried by nitrogen flow, stored overnight under vacuum to remove residual chloroform and finally hydrated in 0.65 ml of 10 mM CHES, 150 mM KCl pH 9 by a 30 min incubation followed by vortex. The lipid suspension was sonicated three to five times during 20 sec each in a Branson-1200 bath sonicator (Danbury, CT). SUV were not stored, they were used the same day upon their preparation.

### Oligomerization of Cyt1Aa and mutant toxins

Oligomerization of Cyt1Aa and mutants was performed as previously described^23^. We incubate 200 ng of Cyt1Aa or Cyt1Aa mutants solubilized protoxin with 90 μl SUV and 10 ng of trypsin in a final volume of 100 μl (2 h, 30 °C, 350 rpm). One mM PMSF (final concentration) was added to stop the reaction. The membrane pellet was separated from the supernatant by centrifugation (30 min, 55,000 rpm). Samples were heated at 65 °C for 3 min, and loaded in SDS-PAGE gels. Proteins were transferred to PVDF Immobilon-P Millipore membranes using a wet chamber (12 h, 150 mA, 4 °C) and revealed by western blot assay as described^23^, using 5% skimmed milk in PBS for blocking (1 h, room temperature), and two washes (5 min each) with PBS containing 0.1% Tween 20 (PBS-Tween). The PVDF was incubated 1 h, room temperature with polyclonal anti-Cyt1A antibody (1:30,000 dilution in PBS-Tween) washed twice with PBS-Tween (5 min each) and incubated with goat anti-rabbit antibody coupled to horseradish peroxidase (Santa Cruz Biotechnology, Dallas, TX) (1:10000 dilution in PBS-Tween). The peroxidase signal was visualized with SuperSignal chemiluminescent substrate (ECL; Amersham Pharmacia Biotech). Oligomerization assays were done three times using different samples of the Cyt1Aa or mutant toxins. Molecular weight markers were Precision Plus Protein Standards All Blue (Bio-Rad) and molecular masses are indicated in kDa. Supplementary Fig. S3 shows additional images of the oligomerization analysis of CytAa, A59C and A61C proteins.

## Electronic supplementary material

Supplementary Information

## Data Availability

All data generated during this study are included in this published article.
