# Supplementary material for: Susceptible and mCry3A resistant corn rootworm larvae killed by a non-hemolytic Bacillus thuringiensis Cyt1Aa mutant
Source: Sci Rep. 2018 Dec 13;8:17805. doi: 10.1038/s41598-018-36205-6 (PMC6292897; doi:10.1038/s41598-018-36205-6)

Susceptible and mCry3A resistant corn rootworm larvae killed by a non-hemolytic *Bacillus thuringiensis* Cyt1Aa mutant.

Alejandra Bravo[1]*, Jazmin A. López-Diaz[1], Takashi Yamamoto[2], Kathleen Harding[2], Jian-Zhou Zhao[3,] Gretel Mendoza[1], Janette Onofre[1], MaryCarmen Torres[1], Mark E Nelson[3], Gusui Wu[2], Amit Sethi[3], and Mario Soberón[1].

Supplementary figure S1. Complete SDS-PAGE of the Cyt1Aa mutants. All samples were boiled 5 min before loading into the SDS-PAGE and the gel was stained with coomassie brilliant blue.

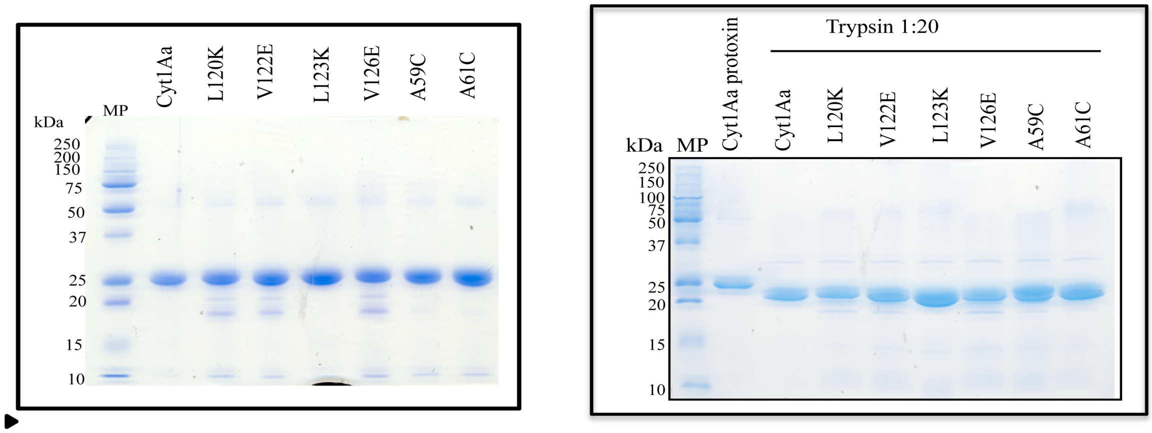

Supplementary figure S2. Addional images of SDS-PAGE analysis of the CytAa, A59C and A61C proteins purified from *B.thuringiensis.* All samples were boiled 5 min before loading into the SDS-PAGE and the gel was stained with coomassie brilliant blue.

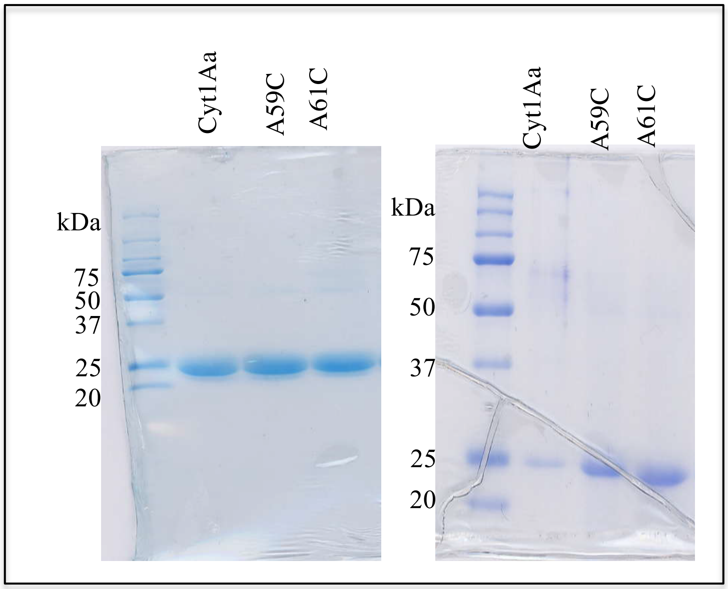

Supplementary figure S3. Addional images of the oligomerization analysis of Cyt1Aa and mutant proteins after activation of solubilized protoxin, with trypsin in the presence of SUV liposomes. Samples were heated 3 min at 65 ºC before loading into the SDS-PAGE transferred to PVDF and reveled in western blot assay as described in materials and methods using polyclonal anti-Cyt1A antibody and goat anti-rabbit antibody coupled to horseradish peroxidase.

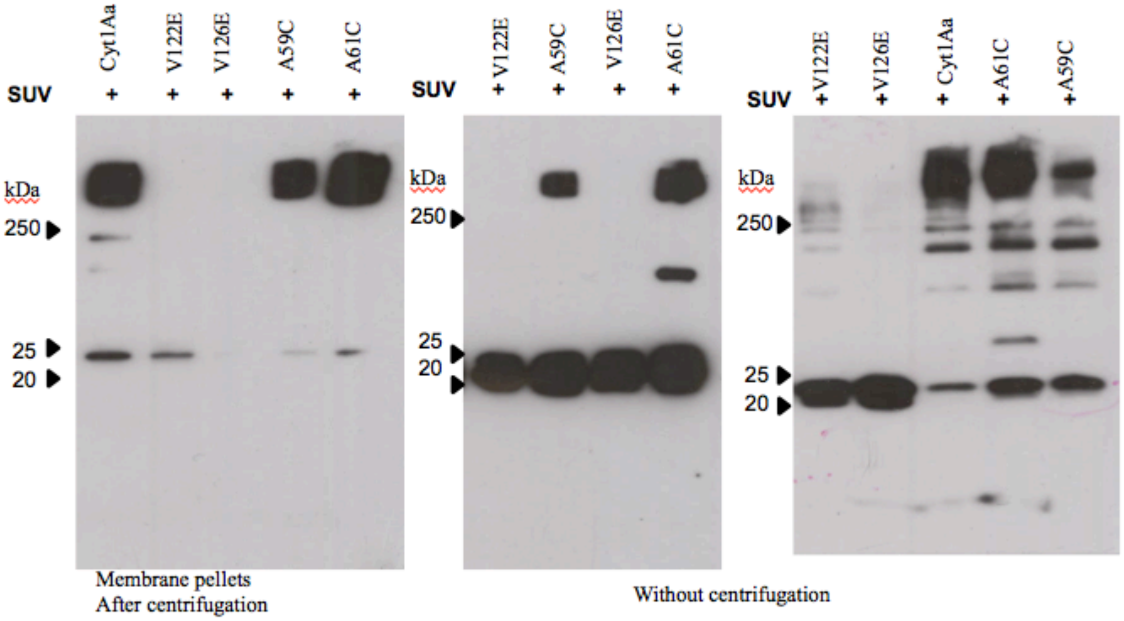

Supplement: Supplementary file 1 — Supplementary Information [file 41598_2018_36205_MOESM1_ESM.pdf]
